# Validation of +tiDx: a point-of-care diagnostic system for Gram-positive bovine mastitis

**DOI:** 10.3389/fvets.2026.1766686

**Published:** 2026-02-11

**Authors:** Valentina Restrepo-Cano, Arley Caraballo-Guzmán, Miryan Margot Sánchez-Jiménez, Giovanny Torres-Lindarte

**Affiliations:** Instituto Colombiano de Medicina Tropical, Universidad CES, Sabaneta, Colombia

**Keywords:** detection, Gram-positive bacteria, identification, intramammary infection, microbiological techniques, on-farm, pathogens

## Abstract

**Introduction:**

Rapid and accurate detection and identification of bovine mastitis-causing pathogens are crucial for treatment decisions and dairy farm management. In this work, we developed and validated a diagnostic system for point-of-care applications that may properly detect and identify *Staphylococcus aureus, Streptococcus agalactiae*, non-*aureus* staphylococci, and other *Streptococcus* species, which are all considered as relevant pathogenic species for bovine mastitis in Colombia. The diagnostic system is supported by a mobile application, +tiApp, that facilitates result interpretation, digital record keeping, and provides evidence-based treatment recommendations to assist on-farm decisions.

**Methodology:**

A total of 520 milk samples from cows with somatic cell count (SCC) < 200,000 cells/ml, or < 100,000 cells/ml for primiparous cows, which were deemed free of intramammary infection, and subclinical mastitis cows (>200,000 cells/ml, or >100,000 cells/ml for primiparous cows) were analyzed using the +tiDx system and a composite reference standard (CRS), which included standard microbiological culture (SMC) and SCC as testing conditions, to assess diagnostic performance for detection and identification of Gram-positive pathogens. We evaluated our diagnostic system by REASSURED criteria against the SMC.

**Results:**

The +tiDx diagnostic system demonstrated adequate overall performance relative to CRS for the detection of mastitis caused by Gram-positive bacteria, achieving a sensitivity of 0.98 and a specificity of 0.94 relative to CRS. Regarding its performance relative to CRS against specific pathogens, the system yielded varied yet noteworthy results depending on the evaluated species: a sensitivity of 0.65 and specificity of 0.94 for *S. aureus*; 0.82 sensitivity and 0.93 specificity for *S. agalactiae*; 0.36 sensitivity and 0.98 specificity for non-*aureus* staphylococci; and values of 0.98 sensitivity and 0.92 specificity for other *Streptococcus* species. Cohen's kappa coefficient was 0.92, indicating high concordance between the in-laboratory diagnostic system and CRS, while concordance between the on-field diagnostic system and CRS was 0.74.

**Conclusion:**

+tiDx is a practical and reliable on-farm diagnostic tool for Gram-positive intramammary infections, combining timeliness and ease of use with appropriate user training to ensure accurate result interpretation and support effective mastitis control.

## 1 Introduction

Mastitis is a major inflammatory condition of the bovine mammary glands, predominantly resulting from intramammary infections (IMI) (1). As the most economically detrimental disease in the dairy industry, mastitis leads to reduced milk production, increased veterinary costs, antibiotic use, and premature culling (2). In 2018, mastitis-associated economical losses in the Bogotá plateau (Colombia) were estimated at USD 70 per cow per year (3). Nonetheless, considering that the department of Antioquia has the largest dairy production in the country, with approximately 17,000 specialized farms and a herd of 133,855 milking cows, losses could exceed previous estimations (4).

Mastitis is primarily associated with bacterial pathogens, including Gram-positive bacteria such as *Staphylococcus* spp. and *Streptococcus* spp., along with Gram-negative microorganisms, mycoplasmas, yeasts, and molds (5). In Colombia, *Staphylococcus aureus, Streptococcus uberis*, and *Streptococcus agalactiae* are the most identified pathogens responsible for the disease (6–8). Given the diverse range of microorganisms that may be involved in the appearance of IMI, accurate identification is essential for effective animal treatment and management (9). Pathogens differ considerably in terms of infection severity and duration, required length of antibiotic treatment, and prevalence. For instance, infections caused by *S. aureus* are often chronic and difficult to treat due to the bacterium's ability to evade the host's immune response and antibiotic therapy (tolerance or resistance) (10), whereas *Escherichia coli* infections tend to be more acute, with most of them resolving spontaneously supported by anti-inflammatory treatment, frequent milking, and fluid therapy, or with standard antibiotic intervention (11, 12).

Basic diagnostics for IMI can be conducted via detection of an increase in somatic cells in milk samples (13). However, in some cases, particular pathogens such as *S. aureus* can evade a host's immune system and prevent an inflammatory response (10, 14), which causes infections to remain undetected due to low somatic cell count (SCC) results. Also, SCC does not allow the identification of the pathogen involved. Because of this, other diagnostic tools could be employed concurrently with SCC analyses, such as microbiological culture or polymerase chain reaction (PCR) (13). Microbiological culture is considered a key component of the diagnostic process, as it facilitates the identification of the pathogen responsible for the infection; however, it requires suitable facilities, laboratory conditions to ensure aseptic handling and minimize contamination, and skilled personnel for accurate on-farm implementation (15). In addition, diagnostic sensitivity (Se) can vary greatly for IMI detection, ranging from 1.6 to 90.4%, depending on the specific pathogen and parameters for positive sample classification (16). Still, its application can aid in the selection of treatment protocols once a pathogenic agent is identified. PCR provides more rapid results than the previous technique and has been shown to provide higher-Se detections when compared to microbiological culturing; however, it requires substantial financial investment which limits its accessibility, pathogen detection is limited to target species included in pre-defined target sets, and DNA traces from contaminants or dead bacteria can be detected and assumed as causative agents for IMI (17). These diagnostic challenges have prompted a reliance on empirical management and treatment strategies, often leading to the implementation of broad-spectrum antibiotic usage which contributes to the growing concern of antimicrobial resistance (AMR) (18). Swift and accurate pathogen identification is therefore critical for both effective treatment and the reduction of AMR (19, 20).

In Colombia, the absence of regulations to control the use of antimicrobial agents in livestock has led to the empirical administration of antibiotics without consulting a veterinarian, including those critical for human health, promoting the development of AMR (21). While these therapies can reduce pathogen prevalence, they may also lead to unnecessary antibiotic use in animals, including those that are not affected, or in situations where antimicrobial therapy is not the most judicious management option. Alternatively, selective treatment protocols, where antibiotics are administered only to infected cows or those with higher likelihood of cure, could reduce AMR impact by up to 66% while also decreasing the economic burden associated with antimicrobial treatments (22).

To address the challenges associated with empirical antibiotic use and the lack of accessible diagnostic alternatives, the implementation of point-of-care (POC) diagnostic tools has emerged as a promising solution (23). These tools can provide affordable, rapid, and accurate diagnostic information on-site, enabling timely decision-making in remote locations, while lowering testing costs, and eliminating the need for trained personnel (23). POC tests can be designed to detect pathogens at varying diagnostic levels, from initial IMI detection to species-level identification (1). Studies have demonstrated that the use of these diagnostic tools can reduce antimicrobial treatments by up to 40% (24), potentially leading to significant reductions in treatment costs for dairy farms, as well as a decrease in AMR due to a reduction is antimicrobial use over selective cow treatment.

Our objectives with this work were to develop and validate a POC diagnostic system based on the detection and identification of the main bacterial pathogens associated with IMI in dairy herds. We hypothesized that the developed system, which we termed +tiDx, would accurately detect and identify *S. aureus, S. agalactiae*, non-*aureus* staphylococci (NAS), and *Streptococcus* spp., thus providing a reliable diagnostic alternative that may aid in decision-making in dairy farms.

## 2 Materials and methods

### 2.1 Development of the +tiDx components

#### 2.1.1 Microcultures

**Figure 1:**
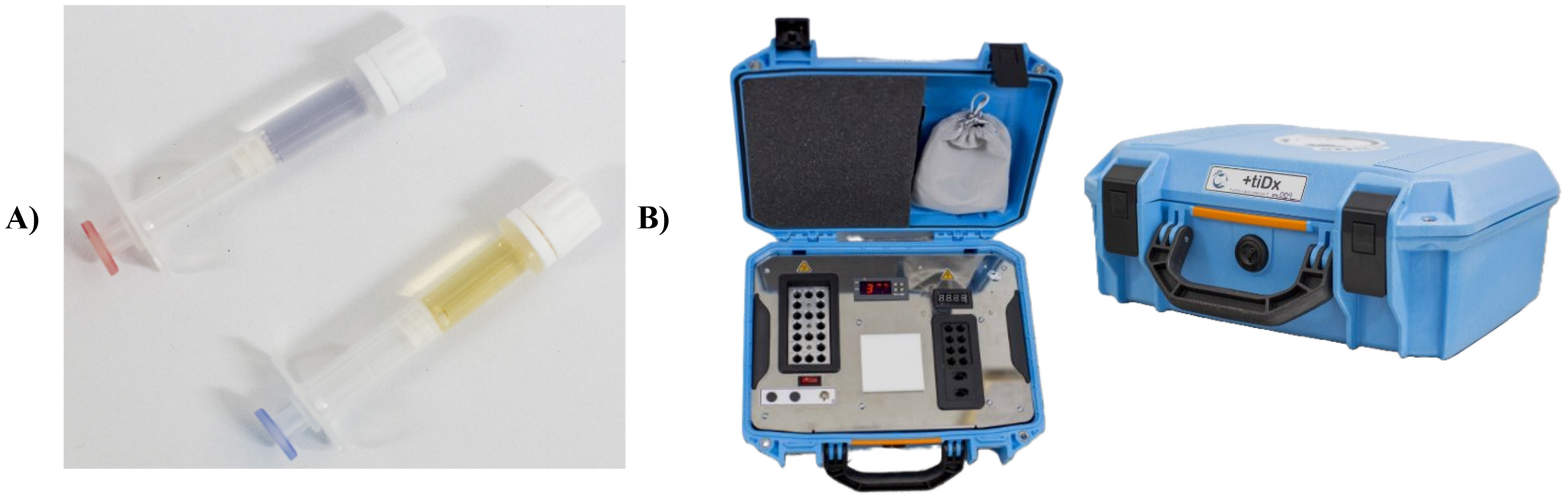
Components of the +tiDx diagnostic system. **(A)** M-GP (blue plunger) and M-GN (red plunger) microcultures; **(B)** Incubator showing incubation module, work module, acrylic plate, and temperature control screen.

A system referred to as “microculture” was developed, consisting of two small syringe-like devices, pre-filled with 400 μl of a specific selective media formulated to support the growth of either Gram-positive (M-GP) or Gram-negative bacteria (M-GN; Figure 1A). The devices were designed to minimize risk of contamination as samples can be directly inoculated from the sample collection container, reducing exposure to environmental microorganisms that could influence results. Bacterial growth in each device was interpreted according to the following criteria: (i) A color change in the M-GN culture media from gray to pale yellow after 24 h of incubation at 37 °C indicated the growth of Gram-negative bacteria. (ii) The interpretation of M-GP required an additional step post-incubation, which involved mixing one drop of M-GP content with one drop of RevelaC, a reagent used for pH-based colorimetric detection of microbial growth. The color transition from blue to yellow confirmed the growth of Gram-positive bacteria.

#### 2.1.2 CromoSyS identification system

This system is based on two commercial chromogenic culture media, named in this study as CromoStaphy (the base culture medium is CHROMagar^TM^ Staph aureus, Paris, France) and CromoStrep (the base culture medium is CHROMagar^TM^ StrepB, Paris, France), which allowed to classify mastitis-associated bacterial pathogens in four categories, including *Staphylococcus aureus* and *Streptococcus agalactiae*, as well as NAS and other *Streptococcus* species. Qualitative interpretation of chromogenic media was performed according to the indication of the manufacturer. On CromoStaphy, *S. aureus* colonies develop a pink to mauve color after incubation, whereas NAS colonies are blue or colorless. Similarly, *S. agalactiae* colonies on CromoStrep are mauve in color after incubation, and colonies of other *Streptococcus* species may appear blue or colorless. It is important to note that in mixed cultures, combinations of colors may also be observed in each medium used (Table 1).

**Table 1:** Algorithm for reading and interpretation of CromoSyS pathogen identification system.

**Result**	**M-GP result**	**M-GN result**	**CromoStaphy result**	**CromoStrep result**
*Staphylococcus aureus*	Growth	No growth	Mauve	Mauve
*Streptococcus agalactiae*	Growth	No growth	Blue OR colorless	Mauve
*Streptococcus* spp.	Growth	No growth	Blue OR colorless	Blue
Non-*aureus* staphylococci	Growth	No growth	Colorless	Colorless
*S. aureus/S. agalactiae*	Growth	No growth	Mauve AND blue	Mauve
*S. aureus/Streptococcus* spp.	Growth	No growth	Mauve OR mauve AND blue	Blue OR mauve AND blue
*S. agalactiae/Streptococcus* spp.	Growth	No growth	Colorless OR blue	Mauve AND blue
Gram-negative	No growth	Growth	N/A	N/A
Contaminated culture	Growth	Growth	N/A	N/A
Negative culture	No growth	No growth	N/A	N/A

N/A, not applicable according to +tiDx algorithm; M-GP, microculture for Gram-positive; M-GN, microculture for Gram-negative; OR, This means that there may be one option or the other, but not necessarily both; AND, This means that both options are present simultaneously.

#### 2.1.3 Incubator

The developed device is composed of two main components which include an incubation module and a workstation (Figure 1B). The incubation module ensures a stable temperature of 37 °C ± 0.5 °C with an independent battery life of up to 10 h after full charge to promote bacterial growth in M-GP and M-GN microcultures, while the workstation provides an area for the set-up of the CromoSyS identification system and result interpretation.

#### 2.1.4 +tiApp mobile application

A mobile application was developed to facilitate bacteriological diagnostics using +tiDx, as the system was designed to be implemented without requiring trained personnel or specific previous qualifications. A culture module found in +tiApp directs the user through a series of questions that are to be answered based on the observations made at each stage of the +tiDx analysis, which include the colors observed during the reading of the microcultures and CromoSyS system. According to the information entered into the application, +tiApp generates the following results options: “*S. aureus*,” “NAS,” “*S. agalactiae*,” “Other *Streptococcus* spp.,” “Gram-negative bacteria,” “*S. aureus*/*S. agalactiae*,” “*S. aureus*/Other *Streptococcus* spp.,” “*S. agalactiae*/Other *Streptococcus* spp.,” “Negative culture,” or “Contaminated culture” (Table 1). In addition, +tiApp centralizes and analyzes a herd's health with relation to bovine mastitis through record-keeping (i.e., SCC, prevalence and type of mastitis, pathogens, administrated antibiotics), providing management and antibiotic treatment recommendations based on identified pathogens and type of mastitis, even in areas with limited internet connectivity, curated by a team of veterinary experts.

### 2.2 Prototype validation

#### 2.2.1 Technical validation at laboratory level

**Figure 2:**
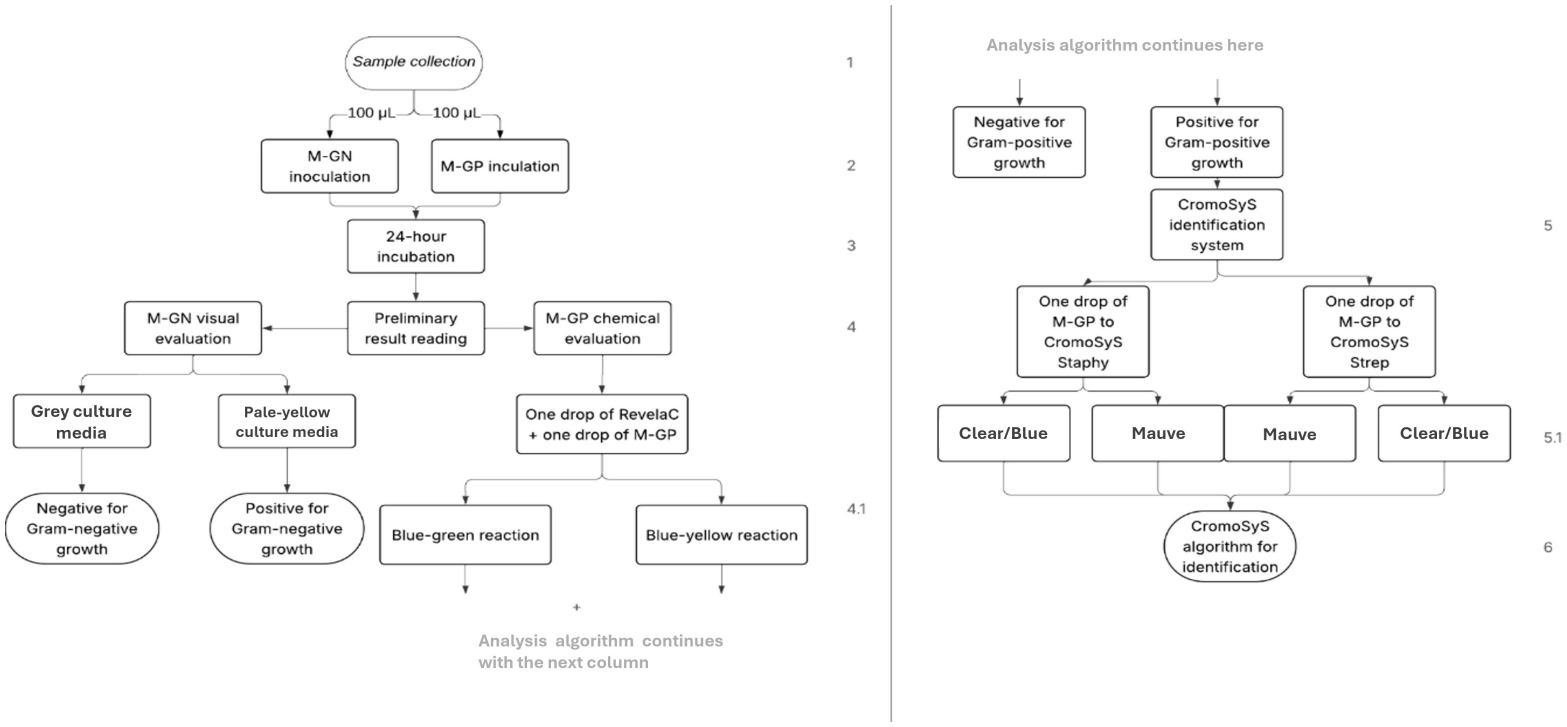
Proposed algorithm for +tiDx analysis based on protocol proposed for detection and identification of mastitis-associated bacterial pathogens. Steps numbers are marked on the right of the diagram.

The technical validation was conducted to assess whether the device produced appropriate results for the intended purpose under laboratory conditions. We evaluated the functionality of +tiDx by inoculating ultra-high temperature (UHT) commercial milk (Leche entera UHT, Colanta, Colombia) and raw bovine milk samples collected from cows with SCC results < 200,000 cells/ml, or < 100,000 cells/ml for primiparous cows, with different concentrations of several reference bacterial strains (American Type Culture Collection—ATCC), either individually or in combination (Table 2), as well as non-contaminated samples (negative control). These samples were analyzed under the proposed +tiDx protocol (Figure 2) and standard microbiological culture (SMC) suggested by National Mastitis Council (NMC) (5). Three replicates were performed for each experiment.

**Table 2:** Pathogen combinations for UHT and raw milk sample evaluation under a controlled contamination model for the determination of limit of detection, and establishment of M-GP and M-GN selectivity and exclusivity.

**M-GP**				**M-GN**			
**Pathogen concentration 10**^2^ **CFU/ml**		**Pathogen concentration 10**^3^ **CFU/ml**		**Pathogen concentration 10**^2^ **CFU/ml**		**Pathogen concentration 10**^3^ **CFU/ml**	
**Main pathogen**	**Mixed pathogens**	**Main pathogen**	**Mixed pathogens**	**Main pathogen**	**Mixed pathogens**	**Main pathogen**	**Mixed pathogens**
*S. aureus*	*E. coli*	*S. aureus*	*E. coli*	*E. coli*	*S. aureus*	*E. coli*	*S. aureus*
	*K. pneumoniae*		*K. pneumoniae*				
	*P. aeruginosa*		*P. aeruginosa*				
*S. epidermidis*	*E. coli*	*S. epidermidis*	*E. coli*				
	*K. pneumoniae*		*K. pneumoniae*		*S. agalactiae*		*S. agalactiae*
	*P. aeruginosa*		*P. aeruginosa*				
*S. agalactiae*	*E. coli*	*S. agalactiae*	*E. coli*				
	*K. pneumoniae*		*K. pneumoniae*				
	*P. aeruginosa*		*P. aeruginosa*		*S. uberis*		*S. uberis*
*S. uberis*	*E. coli*	*S. uberis*	*E. coli*				
	*K. pneumoniae*		*K. pneumoniae*				
	*P. aeruginosa*		*P. aeruginosa*				

Samples were evaluated by +tiDx and standard microbiological culture to evaluate proper microbial growth. M-GP, Gram-positive microculture; M-GN, Gram-negative microculture; *S. aureus* ATCC 6538; *E. coli* ATCC 8739; *Klebsiella pneumoniae* ATCC 13883; *Pseudomonas aeruginosa* ATCC 9027; *Staphylococcus epidermidis* ATCC 12228; *S. agalactiae* ATCC 27956; *S. uberis* ATCC 2795.

##### 2.2.1.1 +tiDx analysis protocol

We followed our proposed analysis and interpretation protocol shown in Figure 2 and Table 1. Briefly, each sample was inoculated into M-GP and M-GN microcultures (Table 2) and incubated for 24 h at 37 °C in the +tiDx incubator device. The microcultures were calibrated to aspirate a previously standardized sample volume of 100 μl. Once the incubation time was completed, bacterial growth was determined in each microculture according to protocol. If an M-GP was positive, one drop (~35 μl) of it was dispensed into each tube of the CromoSyS identification system, which were incubated for 24 h at 37 °C in the incubator device. After incubation time, the CromoSyS was interpreted with the assistance of +tiApp, according to the colors observed in each tube (Table 1). To maintain objectivity, evaluators of the +tiDx protocol were blinded to sample identities and simultaneous SMC results.

##### 2.2.1.2 Standard microbiological culture analysis protocol

To validate the +tiDx results we performed simultaneous analyses using SMC, which we considered as a standard test for result comparison in the technical validation phase, given that SMC is the most used protocol in dairy farms in Antioquia for detection and identification of microorganisms in milk specimens. Ten μL of each milk sample (contaminated and uncontaminated samples of UHT and raw milk) was streaked onto blood-esculin agar (Scharlab, Sentmenat, Spain) and MacConkey agar (Scharlab, Sentmenat, Spain). The inoculated plates were incubated for 24–48 h at 37 °C. Following incubation, bacteriological cultures were interpreted considering the morphological and biochemical (e.g. esculin hydrolysis, hemolysis, Gram staining, catalase reaction) characteristics of the isolated colonies. Presumptive colonies of *S. aureus*, NAS, *S. agalactiae*, and *Streptococcus* spp. were subsequently sub-cultured onto their respective chromogenic agars (CromoStaphy or ChromoStrep) for preliminary identification. Final confirmation of *S. aureus* and *S. agalactiae* was performed using the Vitek^®^ 2 system (bioMérieux, Marcy-l'Étoile, France) for automated pathogen identification. As with +tiDx analysis, evaluators of the SMC were blinded to sample identities and simultaneous +tiDx results.

#### 2.2.2 Diagnostic validation in a relevant environment

##### 2.2.2.1 Sample collection

Milk samples were collected following NMC protocol (https://www.nmconline.org/wp-content/uploads/2016/09/Procedures-for-Collecting-Milk-Samples.pdf) from cows with subclinical mastitis according to SCC results (>200,000 cells/ml, or >100,000 cells/ml for primiparous cows), as well as cows with SCC < 200,000 cells/ml, or < 100,000 cells/ml for primiparous cows, according to the most recent SCC evaluation (performed the same month as sample collection), along with no physical signs and symptoms of infection, such as redness and swelling to the udder or observable changes in milk production. Individual quarter samples as well as composite samples were included in the study (5, 25). Milk samples were collected between May and November 2024 by local personnel from dairy farms located in five municipalities from the northern (San Pedro de los Milagros, Santa Rosa de Osos y Entrerríos) and eastern (La Ceja y Rionegro) regions of the department of Antioquia, Colombia (Supplementary Figure 1). To be included in the study, dairy farms were required to meet three criteria: (i) be located in one of the five municipalities where the study was conducted; (ii) participate in a dairy herd improvement program; (iii) meet the size requirement, as farms were classified based on their herd size as small (from 1 to 50 cows, *n* = 6), medium (from 51 to 100 cows, *n* = 8), or large (> 100 cows, *n* = 6). According to these criteria, 20 dairy farms were included. The number of milk samples collected from each farm was determined by convenience sampling, based on the mastitis prevalence reported by the farms and considering the minimum sample size recommended by the World Organization for Animal Health for the validation of diagnostic tests for infectious diseases (26). Based on an expected diagnostic Se and diagnostic specificity (Sp) relative to SMC of 95%, a 95% confidence level, and a 2% margin of error, the recommended sample size was 456 milk samples. All data collection was planned prior to sample analysis.

##### 2.2.2.2 Sample analysis

Each dairy farm was provided with +tiDx kits, which included a portable incubator device, M-GP and M-GN microcultures, CromoSyS vials, RevelaC reagent vial, disposable plastic pipettes with a capacity of 200 μl, mixing sticks, and instructions with explanatory videos for all procedures involved. In addition, local farm employees were trained in +tiDx analysis and result interpretation by accompanying veterinary personnel throughout the validation process, along with proper milk sampling practices based on recommendations by NMC (https://www.nmconline.org/wp-content/uploads/2016/09/Procedures-for-Collecting-Milk-Samples.pdf). Briefly, farm employees were instructed to thoroughly clean and disinfect the udder prior to sample collection and discard several streams of milk from the teat before collecting the necessary specimen in a sterile vial and refrigerating it until time of analysis. Milk samples were collected in duplicate as counter-samples. One sample was analyzed on-farm using +tiDx, while the counter-sample was refrigerated and subsequently transported to the Industrial Microbiology Laboratory of the Colombian Institute of Tropical Medicine-CES University for processing by SMC and +tiDx, in accordance with the established protocols (Sections 2.2.1.1 and 2.2.1.2). Samples were intended to be transported and analyzed on the same day as collection. However, there were instances where samples were evaluated the following day due to logistic complications. Still, all samples were evaluated within 24 h of collection. The positive M-GP analyzed at the Colombian Institute of Tropical Medicine laboratory were sub-cultured on blood-esculin agar and MacConkey agar plates (in this media to confirm selectivity) for bacterial isolation, which were subsequently classified by chromogenic agars. Milk samples analyzed at laboratory level were processed by microbiologists with previous experience in mastitis diagnostics. Laboratory results from both +tiDx and SMC evaluations were not available to readers of field-evaluated samples.

### 2.3 Statistical analysis

Data collection was planned before testing was performed and analyses were conducted separately for the validation phases. For the technical phase, we calculated concordance between +tiDx and SMC (C1), intermediate precision, limit of detection (LOD), analytical Sp (selectivity, exclusivity), diagnostic Se and diagnostic Sp. For the diagnostic validation, concordance between in-laboratory +tiDx and a defined composite reference standard (CRS;C2), concordance between on-field +tiDx results and CRS (C3), concordance between in-laboratory and on-field +tiDx analysis (C4), and concordance between a subset of in-laboratory and on-field +tiDx analysis (C5) were calculated, as well as diagnostic Se and diagnostic Sp relative to CRS.

Microbiological culture is an imperfect reference standard, with higher diagnostic Sp than diagnostic Se. We recognized its limited diagnostic Se as a methodological constraint and, therefore, a CRS was employed. The CRS integrated SMC and SCC (with higher diagnostic Se than diagnostic Sp, and the most common screening tool for IMI in Colombia). A parallel testing approach was implemented, in which a sample is considered positive when at least one of the two tests is positive. A positive SMC result was defined as the presence of ≥10 presumptive CFU/0.01 mL of at least one recognizable pathogen, according to the NMC protocol and the definition for classifying quarters affected by IMI based on Dohoo et al. (5, 16). For the SCC, a sample was considered positive when SCC exceeded 200,000 cells/ml in multiparous cows or 100,000 cells/ml in primiparous cows (5), and negative when below these thresholds. Based on these criteria, a sample was classified as “Positive relative to the CRS (P-CRS)” when it met at least one of the following operative conditions: +tiDx identified the same bacterial species detected by SMC with ≥10 CFU/0.01 ml growth, or SCC exceeded 200,000 cells/ml in multiparous cows or 100,000 cells/ml in primiparous cows. By contrast, a sample was classified as “Negative relative to the CRS (N-CRS)” only when all of the following conditions were met: +tiDx showed no growth (see Table 1), SMC showed no growth or < 10 CFU/0.01 ml, and SCC was < 200,000 cells/ml in multiparous cows or < 100,000 cells/ml in primiparous cows. This parallel approach maximizes the Se of the CRS. On the other hand, “False positives relative to the CRS (FP-CRS)” were defined as cases in which +tiDx detected a pathogen different from the one identified by SMC with ≥10 CFU/0.01 ml, or SCC was below the established threshold. “False negatives relative to the CRS (FN-CRS)” were defined as samples with no growth in +tiDx but with ≥10 CFU/0.01 ml growth in SMC or SCC above the defined threshold. A sample was considered contaminated when SMC isolated three or more distinct colony types. In cases when a precise verdict on presence or absence of infection or identification of such could not be reached, or samples were compromised in any way, specimens were removed from statistical analysis. Based on these definitions, 2 x 2 tables were constructed for the estimation of concordance, intermediate precision, diagnostic Se and diagnostic Sp relative to CRS.

For the technical validation phase, C1 was determined by comparing results from milk samples (*n* = 180) evaluated using the +tiDx diagnostic system against those obtained from the SMC. These samples were also used for the calculation of diagnostic Se and diagnostic Sp, which we defined as the proportion of positive results which matched the results obtained through SMC analysis, and the proportion of negative results which matched the results obtained through SMC analysis, respectively.

Intermediate precision (*n* = 360) was assessed by the calculation of a Cohen's kappa coefficient for repeated measurements conducted by two independents microbiologists with comparable levels of proficiency. This analysis was performed to provide an estimate of the concordance between repeated tests under analyst variation, measuring the reliability of the +tiDx prototype for IMI detection. The kappa coefficient was interpreted based on standard guidelines (27):

- < 0.00: poor concordance.
- 0.00–0.20: slight concordance.
- 0.21–0.40: fair concordance.
- 0.41–0.60: moderate concordance.
- 0.61–0.80: substantial concordance.
- 0.81–1.00: perfect concordance.

LOD (*n* = 480) was established as the lowest concentration of pathogen that could be reliably detected by the diagnostic system. This was assessed by the evaluation of two different concentrations (10^2^ CFU/ml and 10^3^ CFU/ml) of several pathogens (*S. aureus, S. epidermidis, S. agalactiae, S. uberis, E. coli*, and *K. pneumoniae*) for both M-GP and M-GN microcultures (Table 2). These assays were performed by two different microbiologists.

Selectivity was measured by assessing the M-GP and M-GN microcultures' ability to accurately promote the growth of Gram-positive and Gram-negative bacteria, respectively, while exclusivity was assessed by the ability of the identification system to differentiate among *Staphylococcus* and *Streptococcus* species accordingly (Table 2). Each microculture and CromoSyS was inoculated with Gram-positive (*S. aureus, S. epidermidis, S. agalactiae, S. uberis*) and Gram-negative (*E. coli, K. pneumoniae*, and *Pseudomonas aeruginosa*) bacteria at two different concentrations (10^2^ CFU/ml and 10^3^ CFU/ml). Samples (*n* = 162) were incubated for 24 h at 37 °C, and the microcultures' selectivity and CromoSyS' exclusivity was verified by recovery of the specific bacteria (Gram-positives in M-GP and Gram-negatives in M-GN) or growth inhibition (Gram-positives in M-GN and Gram-negatives in M-GP) and identification differentiation in the chromogenic media.

For the diagnostic validation, C2 was defined as the degree to which +tiDx analysis performed in-lab produced the same results as those obtained by CRS. We also calculated the degree of difference in results obtained through sample evaluation by +tiDx on the field and CRS within laboratory settings (C3), from in-laboratory and on-farm +tiDx results (C4), and in-laboratory and on-farm results from a subset of samples (*n* = 95; C5), corresponding to samples collected and processed by the researchers in dairy farms at the same time as regular on-farm +tiDx evaluations were performed. All concordance values (C1–C5) were quantified using Cohen's kappa coefficient.

**Figure 3:**
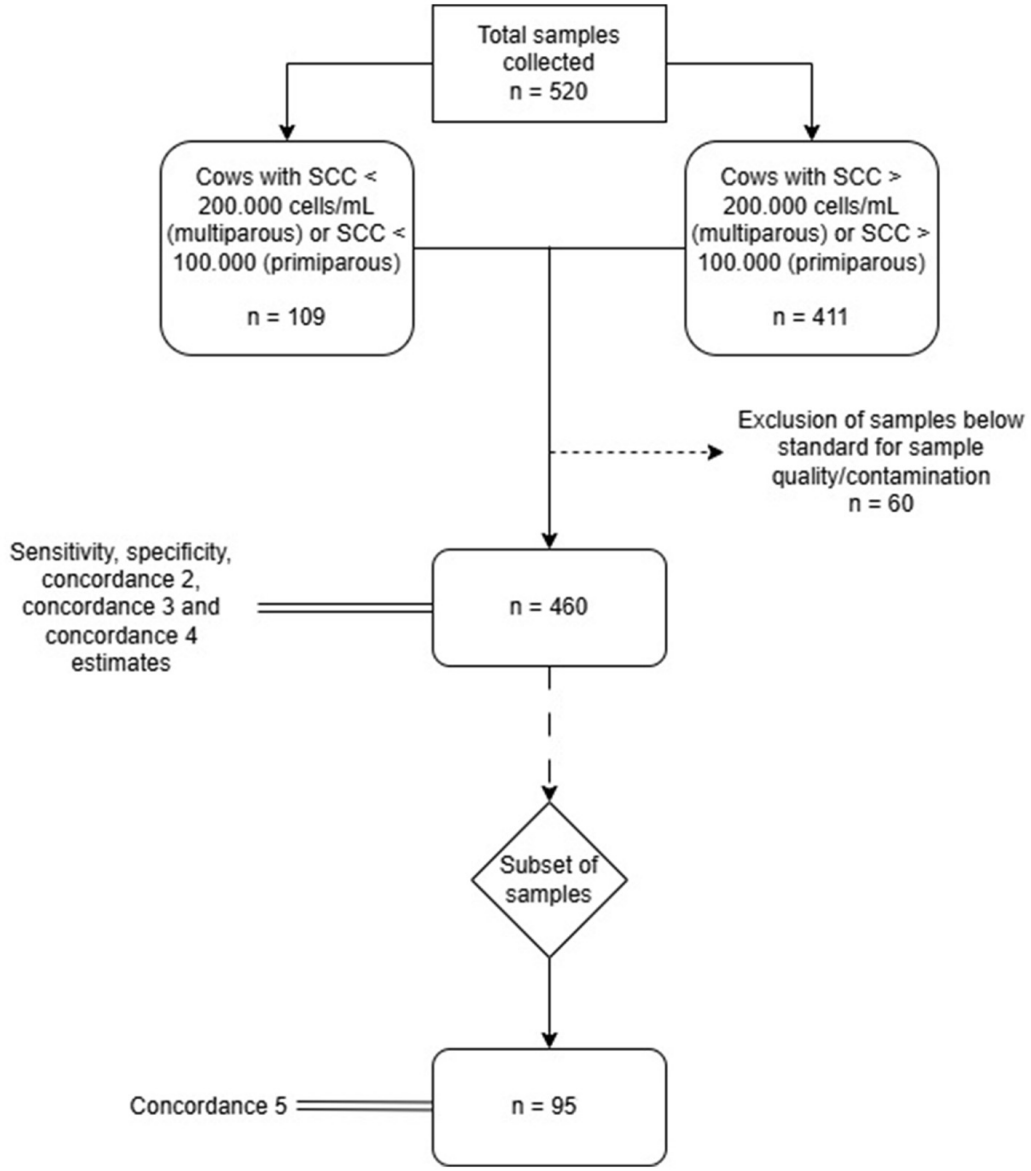
Flow of sample inclusion and distribution in the diagnostic validation phase. Straight arrows indicate flow of chart and inclusion of samples. Dashed arrows indicate sample exclusion. Double lines indicate process association to sample amount.

Diagnostic sensitivity relative to CRS was defined as the proportion of P-CRS results out of all positive cases declared by CRS, and diagnostic specificity relative to CRS was defined as the proportion of N-CRS results out of all negative cases declared by CRS. For all calculated variables, 95% confidence intervals (CI) were provided to indicate the precision of estimation. Analyses were performed in RStudio (28) supported by the EpiR package (29). Evaluators were blinded to simultaneous test results to prevent bias. Flow of sample inclusion is shown in Figure 3.

### 2.4 REASSURED comparison

We used the REASSURED criteria proposed by Land et al. (30) to evaluate and compare the developed prototype to SMC. We defined numerical values for each of the REASSURED criteria [***R**eal-time connectivity*, ***E**ase of specimen collection*, ***A**ffordable*, (Diagnostic)] ***S**ensitivity, [*(Diagnostic) ***S**pecificity*, ***U**ser-friendly*, ***R**apid & Robust*, ***E**quipment-free, and* ***D**eliverable*] and determined a scale with which we could evaluate both prototype and SMC. It is important to note that the criteria and descriptions of the scale were tailored to the conditions commonly found in small and medium dairy farms in Colombia. We calculated the overall REASSURED score of either diagnostic strategy by assigning equal weights to each criterion.

- *Real-time connectivity* was evaluated as to whether the tests were associated with a reader or a mobile device that could read and/or interpret the test result, and aid in providing information for decision-making.
- *Ease of specimen collection* was regarded as whether the tests were designed to require non-invasive specimens.
- *Affordable* was defined as the average cost per sample tested, considering the diagnostic context and information that may be provided by its implementation.
- *Sensitivity* was considered as the capability of the tests, expressed in percentages, to detect infected samples obtained from infected animals
- *Specificity* was considered as the capability of the tests, expressed in percentages, to detect non-infected samples obtained from non-infected animals.
- *User-friendly* was quantified by the number of steps required for sample analysis, as well as whether the test requires qualified or trained personnel (or specific environmental/aseptic conditions) for either the application of the testing process or result reading and interpretation.
- *Rapid & Robust* was regarded as the amount of time necessary for definitive pathogen identification once the sample is collected. Moreover, we compared the transport and storage conditions required for accurate performance.
- *Equipment-free* was evaluated as to whether the testing process required additional equipment, such as readers or incubators, for its application or result analysis. We included considerations for rechargeable, pluggable, and/or battery-operated devices which allow for portability.
- *Deliverable* was estimated by whether the test was accessible to dairy farms. For this, we compared whether the tests could be purchased in local agricultural warehouses.

### 2.5 Ethics statement

This study was reviewed and approved by the Comité Institucional para el Cuidado y Uso de los Animales (CICUA) of Universidad CES under protocol Ae-035 (approved November 2021). All procedures involved only non-invasive collection of milk specimens from lactating dairy cows and were conducted as part of routine farm management. Milk sampling followed the National Mastitis Council protocol “Procedures for Collecting Milk Samples.” Participation of the farm was voluntary, and informed consent was obtained from the farm owners prior to sample collection. All animal handling complied with institutional and national guidelines for the ethical use of animals in research.

## 3 Results

### 3.1 Technical validation at laboratory level

We analyzed a total of 1,182 artificially contaminated milk samples to determine concordance, intermediate precision, LOD, selectivity and exclusivity for M-GP and M-GN microcultures, diagnostic Se relative to SMC, and diagnostic Sp relative to SMC.

We obtained a concordance of 100% (kappa = 1.0; CI 1.0–1.0) between the samples evaluated by SMC under the protocol proposed by NMC (5) and those evaluated using +tiDx. Regarding the diagnostic Sp and diagnostic Se, both relative to SMC, proportions of 1, or 100%, for both Se (Se = 1.0; CI 1.0–1.0) and Sp (Sp = 1.0; 1.0–1.0) were obtained (Table 3). Intermediate precision evaluations demonstrated complete concordance (kappa = 1.0; CI 1.0–1.0) amongst the results obtained by both analysts for all pathogens assessed. We determined an LOD of 10^2^ CFU/ml for all evaluated pathogens, with an exception for *S. epidermidis* for which LOD was determined as 10^3^ CFU/ml. We found that morphological and biochemical assessments revealed a 100% Gram-positive bacteria recovery rate with no growth or interference from Gram-negative bacteria from M-GP microcultures, and a 100% Gram-negative bacteria recovery rate from M-GN microculture, with no growth or interference from Gram-positive microorganisms, as well as a 100% identification differentiation among *Staphylococcus* and *Streptococcus* species.

**Table 3:** Diagnostic performance of M-GP and M-GN microcultures for the detection of mastitis-associated pathogens determined from sample evaluation by +tiDx, and a composite reference standard (CRS) consisting of somatic cell count (SCC) and standard microbiological culture (SMC).

**Variable**	**Result (CI)**
**Technical validation**
Concordance 1^*^	1.0 (1.0–1.0)
Intermediate precision	1.0 (1.0–1.0)
Limit of detection^a^	10^2^ CFU/ml
Selectivity	Gram-positive cocci for M-GP and Gram-negative bacilli for M-GN.
Sensitivity	1.0 (1.0–1.0)
Specificity	1.0 (1.0–1.0)
**Diagnostic validation**
Sensitivity	0.98 (0.95–0.99)
Specificity	0.94 (0.89–0.96)
Concordance 2^†^	0.92 (0.88–0.95)
Concordance 3^‡^	0.74 (0.68–0.80)
Concordance 4^§^	0.54 (0.42–0.66)
Concordance 5^¶^	0.70 (0.55–0.85)

Total number of evaluated samples: 520. ^a^With the exception of S. epidermidis which LOD is 10^3^ CFU/ml; CI, confidence interval; M-GP, Gram-positive microculture; M-GN, Gram-negative microculture. ^*^Concordance 1: concordance between results obtained by +tiDx and SMC evaluation; ^†^Concordance 2: concordance between in-laboratory +tiDx and CRS results; ^‡^Concordance 3: concordance between on-field +tiDx and CRS results; ^§^Concordance 4: concordance between in-laboratory and on-field +tiDx analysis; ^¶^Concordance 5: concordance between a subset of in-laboratory and on-field +tiDx results.

### 3.2 Diagnostic validation

A total of 520 milk samples collected from cows belonging to 20 dairy farms were tested. From 520 samples, 411 were obtained from cows with subclinical mastitis and 109 samples were obtained from cows with SCC < 200,000 cells/ml in multiparous cows or < 100,000 cells/ml in primiparous cows. The number of samples evaluated per farm ranged from 6 to 65 (Table 4). No adverse events occurred on the participating cows from performing either +tiDx or CRS analysis as sample collection is non-invasive.

**Table 4:** Total number of diagnostic samples collected classified by location and size of dairy farm.

**Region**	**Municipality**	**Dairy farm**	**Size**	**Number of cows**	**Number of samples**
North	San Pedro de los Milagros	1	Medium	99	65
		2	Small	33	23
		3	Small	34	13
		4	Large	133	50
		5	Medium	69	11
		6	Large	141	15
	Entrerríos	7	Small	39	24
		8	Medium	66	14
		9	Medium	83	38
		10	Large	246	30
	Santa Rosa de Osos	11	Small	42	6
		12	Medium	77	21
		13	Medium	80	15
		14	Large	123	58
East	Rionegro	15	Small	39	20
		16	Medium	61	26
		17	Large	102	42
		18	Small	10	12
	La Ceja	19	Large	109	19
		20	Medium	95	18
**Total**				1,681	520

Farms were classified based on their herd size as small (from 1 to 50 cows, *n* = 6), medium (from 51 to 100 cows, *n* = 8), or large (>100 cows, *n* = 6).

Out of the 520 samples collected, 33 samples were considered contaminated in accordance with SMC results (≥ three distinct types of isolated colonies) (16) and were excluded from subsequent analyses. Of these, 21 (63.6%) were also considered contaminated by the +tiDx system due to growth on both M-GP and M-GN, as defined in the protocol for this type of samples. Additionally, 27 more samples were discarded because they did not meet the quality criteria for processing, which included spilled samples and violation of cold chain maintenance (ensured freezing/refrigeration, insulated cooler or thermal box, and avoidance of leakage/cross contamination).

Of the remaining 460 samples, 216 were considered positive according to the specifications defined for the CRS, that is, the presence of ≥10 CFU/0.01 ml of at least one pathogen on the inoculated blood-esculin agar culture medium or values over 200,000 cells/ml in multiparous cows or 100,000 cells/ml in primiparous cows. None of the identified pathogens were classified as Gram-negative organisms. No bacterial growth was observed in the remaining 244 samples. In contrast, 251 samples were classified as positive using +tiDx, while 209 were considered negative. No Gram-negative bacteria were identified.

**Figure 4:**
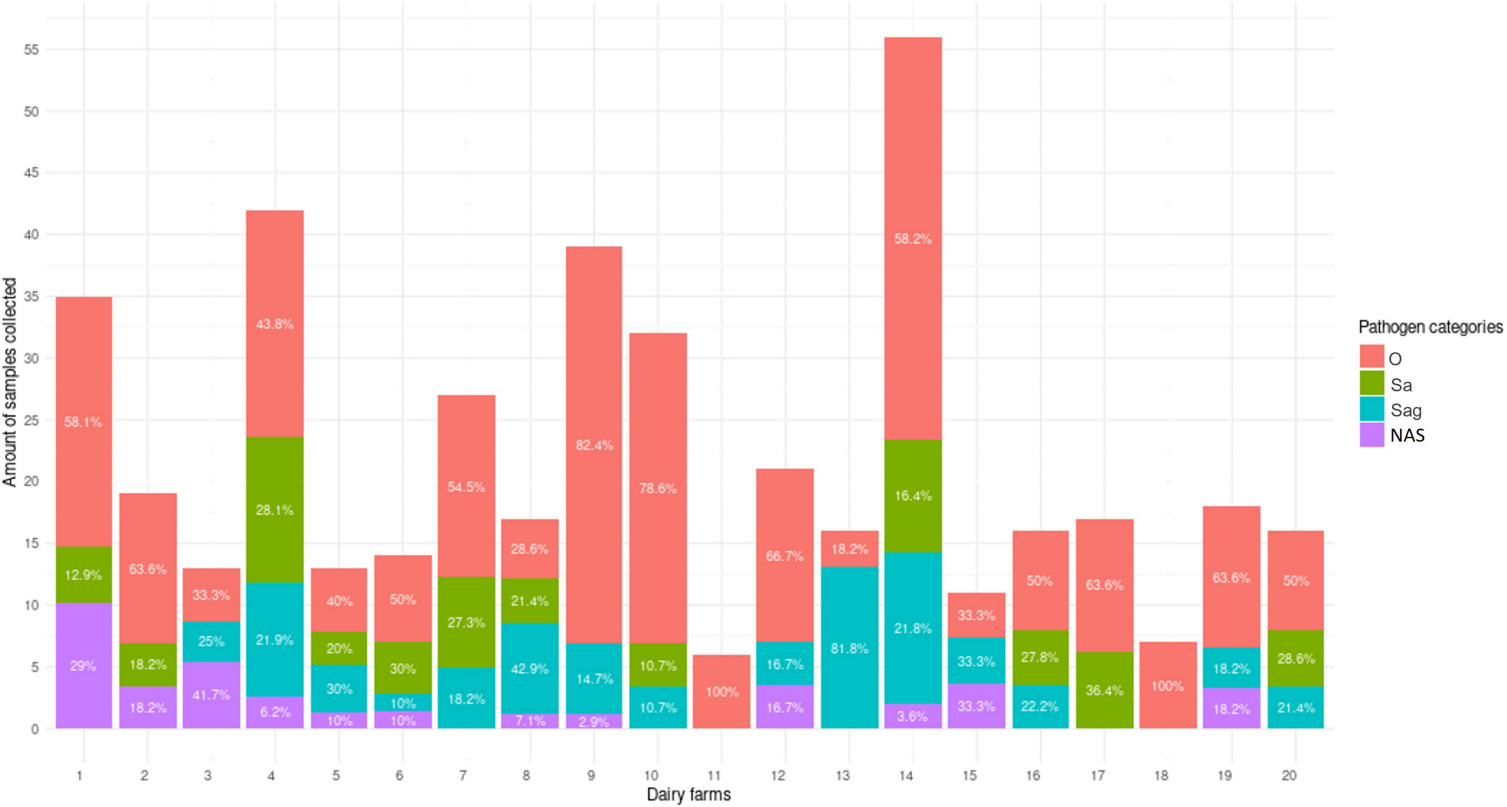
Results derived from positive samples obtained in diagnostic validation phase. *Y*–axis shows the number of samples collected in each dairy farm, and the numbers in each section of the stacked bars represent the percentage of each category of pathogens (O: Other *streptococcal species*; Sa: *S. aureus*; Sag: *S. agalactiae*; NAS: non-*aureus staphylococci*) found in those samples.

Table 5 presents the results obtained by each diagnostic system evaluated (Figure 4). It is important to note that cows from which the samples were collected were not subjected to any clinical interventions that may have affected the results obtained from either CRS or +tiDx testing, as samples and counter-samples for both diagnostic systems were obtained during the same sample collection process.

**Table 5:** Comparison between SMC and +tiDx diagnostic-sample evaluation results.

**Results**	**Standard microbiological culture**	**+tiDx diagnostic system**
**Gram-positive bacteria**	216	251
*S. aureus*	24	25
*S. agalactiae*	25	37
*Streptococcus* spp.^*^	98	133
NAS	20	23
*S. aureus* + NAS	1	NR
*S. aureus* + *S. agalactiae*	2	5
*S. aureus* + *Streptococcus* spp.	16	13
*S. agalactiae* + NAS	7	NR
*S. agalactiae* + *Streptococcus* spp.	10	12
*Streptococcus* spp. + NAS	13	3
**Gram-negative bacteria**	NR	NR
**Negative culture**	244	209
**Contaminated samples**	33	21

Pathogen information was classified according to +tiDx identification algorithm. Total number of evaluated samples: 520. NAS, non-aureus staphylococci; NR, no result was obtained. ^*^This group included species other than *S. agalactiae*. ^+^Indicates a mixed culture. In cases when a precise verdict on presence or absence of infection or identification of such could not be reached, or samples were compromised in any way, specimens were removed from statistical analysis and are not included in these results.

The diagnostic performance was evaluated using the 460 available samples, including concordance between in-laboratory +tiDx and CRS (C2), and between on-farm +tiDx evaluations and CRS (C3), as well as diagnostic Se and diagnostic Sp relative to CRS. Also, we evaluated concordance between +tiDx evaluations performed in-laboratory vs. on-farm (C4). A subset of samples (*n* = 95), corresponding to counter-samples collected and processed by the researchers directly in dairy farms, were also used to determine the same concordance (C5).

The diagnostic system demonstrated a diagnostic Se relative to CRS of 0.98 (0.95–0.99) and diagnostic Sp relative to CRS of 0.94 (0.89–0.96) for IMI detection caused by Gram-positive bacteria, with perfect concordance (kappa = 0.92; CI 0.88–0.95) for C2. Diagnostic performance results for target pathogens are shown in Table 6. Regarding C3, we obtained a substantial concordance between in-laboratory +tiDx and on-farm evaluations (kappa = 0.74; CI 0.68–0.80).

**Table 6:** Diagnostic performance of the +tiDx system relative to composite reference standard for the identification of mastitis-associated pathogens, calculated from 2 x 2 tables.

**Variable**	***S. aureus* (CI)**	***S. agalactiae* (CI)**	**NAS (CI)**	*Streptococcus* spp.^*^(CI)
Sensitivity	0.65 (0.50–0.78)	0.82 (0.69–0.92)	0.36 (0.24–0.49)	0.98 (0.94–0.99)
Specificity	0.94 (0.90–0.97)	0.93 (0.89–0.96)	0.98 (0.96–1.00)	0.92 (0.84–0.97)
Total number of positive samples per +tiDx evaluation	43	54	26	161

Total number of evaluated samples: 520. CI, confidence interval; NAS, non-*aureus* staphylococci. ^*^This group included species other than *S. agalactiae*.

Concordance for the diagnosis of Gram-positive bacteria was moderate (kappa = 0.54; CI 0.42–0.66) for C4 and substantial (kappa = 0.70; CI 0.55–0.85) for C5. Concordance for pathogen identification was substantial for *S. agalactiae* (kappa = 0.73; CI 0.52–0.94), and other *Streptococcus* species (kappa = 0.64; CI 0.44–0.85). In contrast, the concordance was moderate for *S. aureus* (kappa = 0.58; CI 0.32–0.83) and NAS (kappa = 0.45; CI 0.04–0.87).

### 3.3 REASSURED comparison

We compared the +tiDx diagnostic system and the SMC based on the REASSURED criteria (30). See Table 7 for the REASSURED criteria and scoring of each protocol. From a total of 55 possible points, SMC obtained 24 points, 43.6% of all possible points, with Ease of specimen collection and Specific representing its strongest criteria. +tiDx obtained a total of 47 points, 85.4% of all possible points, and its strongest criteria were Real-time connectivity, Ease of specimen collection, Affordability, Sensitive, User-friendly 2, and Deliverable.

**Table 7:** Comparison of +tiDx prototype vs. SMC based on adjusted REASSURED criteria.

**REASSURED criteria**	**Evaluated criteria/score**				**+tiDx score result**	**Standard microbiological culture score result**
**R**eal-time connectivity	Test is associated to a device/external support that facilitates result reading/analysis		Test is not associated to a device/external support that facilitates result reading/analysis		5	1
	5		1			
**E**ase of specimen collection	Test allows the use of non-invasive specimens for analysis		Test does not allow the use of non-invasive specimens for analysis		5	5
	5		1			
**A**ffordable^a^	Price between 1.1 USD and 4.7 USD	Price between 4.8 USD and 9.48 USD	Price between 9.49 USD and 14.22 USD	Price is greater than 14.22 USD	5	3
	5	4	3	2		
**S**ensitive	Test can identify infected samples in 80%−100% of the evaluated samples		Test can identify infected samples in 80% or less of the evaluated samples		5	1
	5		1			
**S**pecific	Test can identify non-infected samples in 80%−100% of the evaluated samples		Test can identify non-infected samples in 80% or less of the evaluated samples		5	5
	5		5			
**U**ser-friendly	The test requires 4–5 steps for pathogen identification	The test requires 6–7 steps for pathogen identification	The test requires 7–8 steps for pathogen identification	The test requires 9 or more steps for pathogen identification	4	2
	5	4	3	2		
	Test requires experienced/trained personnel or specific environmental conditions for its application and result analysis		Test does not require experienced/trained personnel or specific environmental conditions for its application and result analysis		5	1
	1		5			
**R**apid & Robust	Test requires 0–12 h for pathogen identification	Test requires 12–24 h for pathogen identification	Test requires 24–48 h for pathogen identification	Test requires more than 48 h for pathogen identification	3	2
	5	4	3	2		
	Test requires specific storage conditions regarding temperature and humidity for proper functioning		Test does not require specific storage conditions regarding temperature and humidity for proper functioning		1	1
	1		5			
**E**quipment free	Test does not require additional equipment for its application or result reading/analysis	Test requires additional equipment which is rechargeable	Test requires additional equipment which is pluggable/non-portable	Test requires additional equipment which is not included in the test.	4	2
	5	4	3	2		
**D**eliverable	Test may be acquired in local agricultural warehouses		Test must be acquired through specialized distributers		5	1
	5		1			
**Total**	47	24

^a^Ranges were adjusted for Colombian average affordability, test prices and availability.

## 4 Discussion

### 4.1 Validation

Traditional microbiological culture has been widely used for IMI diagnosis. However, as an imperfect test, its performance can vary significantly depending on the type of microorganism involved, sample quality, and the thresholds applied for result interpretation (1 or 10 CFU/0.01 ml) (16). Moreover, the implementation of microbiological culture under field conditions faces limitations related to the need for appropriate infrastructure that meets the requirements for microbiological analysis, as well as the professional training of personnel responsible for its operation (5). These requirements often result in high operational costs, limiting their widespread use in routine diagnostics. Hence, there is a need for POC diagnostic tools, such as the +tiDx system, to facilitate on-farm detection. These tools can support more effective mastitis control strategies and improve antimicrobial use. There is increasing interest within the dairy industry in adopting POC diagnostic devices to guide antimicrobial treatments, amid growing pressure on farmers to minimize antibiotic use (24, 31, 32).

The +tiDx system demonstrated high overall performance for the detection of IMI caused by Gram-positive bacteria, with a diagnostic Se of 0.98 and diagnostic Sp of 0.94, both relative to CRS. A study conducted in Australia evaluated several POC tests for the identification of pathogens causing clinical mastitis and reported varying results regarding the detection of Gram-positive bacteria (15). Although the testing conditions are not entirely comparable, as our study focused on subclinical mastitis cases and we acknowledged SMC as an imperfect standard, the diagnostic Se and diagnostic Sp of +tiDx were higher than those reported for the Check-up (0.89 and 0.79, respectively) and Mastatest (0.55 and 0.81) devices. In the case of Accumast, it showed a similar diagnostic Sp (0.90) yet lower diagnostic Se (0.76) than +tiDx. Another recently developed technology (Vetscan^®^ Mastigram+), based on a flow dipstick test for the detection of Gram-positive mastitis, reported a diagnostic Se of over 0.99 and a diagnostic Sp of 1.0 (33). It is important to note that none of these technologies are available in Colombia. Access to diagnostic tools with appropriate and stable Se and Sp could be essential for guiding selective treatment strategies, focusing only on cows that require it. Adequate diagnostic performance enables informed decisions regarding the presence or absence of Gram-positive IMI prior to the initiation of dry cow therapy (22).

As observed with other culture-based diagnostic tools, +tiDx performance varied depending on the pathogen. For *S. aureus*, diagnostic Se and diagnostic Sp relative to CRS were 0.65 and 0.94, respectively, indicating limited Se but high Sp. In the case of *S. agalactiae*, the system showed a strong diagnostic performance relative to CRS (diagnostic Se = 0.82, diagnostic Sp = 0.93). For NAS, diagnostic Se relative to CRS was low (0.36), while diagnostic Sp relative to CRS remained high (0.98), suggesting that although the test is highly specific, a significant number of infections may go undetected. The highest diagnostic performance was observed for other *Streptococcus* species, with a diagnostic Se relative to CRS of 0.98 and diagnostic Sp relative to CRS of 0.92. This highlights the ability to correctly identify this group, which includes *S. uberis*, one of the most prevalent pathogens in Colombia and other countries (34). Recently, our group published a study highlighting the high genetic heterogeneity of *S. uberis* in Colombia. The study identified10 novel sequence types and detected genes (*tetM, tetO, ermB*) and mobile genetic elements (*repUS43, ISSag2, ISEnfa4*) that have been associated with antimicrobial resistance (34). These findings underscore the epidemiological complexity of *S. uberis* in the region. They also emphasize the need for targeted control and surveillance strategies, such as bacteriological monitoring. One of the culture-based technologies available in Colombia, originally developed in Brazil, showed a diagnostic Se of 0.84 and a diagnostic Sp of 0.96 for the detection of *S. uberis* during its validation study (35). In comparison, Check-up reported values of 0.66 and 0.89, Mastatest 0.31 and 0.94, and Accumast 0.16 and 0.96, respectively (15). In the study by Dohoo et al. (16), depending on the diagnostic definition applied, the Se for detecting *S. uberis* and *Streptococcus dysgalactiae* ranged from 0.67 to 0.87, while Sp remained consistently high, reaching up to 1.0.

In our study, +tiDx showed a diagnostic Se relative to CRS of 0.65 and a diagnostic Sp relative to CRS of 0.94 for detecting IMI caused by *S. aureus*. Similarly, Granja et al. demonstrated slightly higher values, with a diagnostic Se of 0.74 and a diagnostic Sp of 0.98 (35). These results fall within the range reported by Dohoo et al. (16). They found that the diagnostic Se and diagnostic Sp of microbiological culture based on a single milk sample ranged from 0.44 to 0.90 and up to 1.00, respectively. Their estimates varied depending on factors such as number of CFU/0.01 ml (1 or 10 CFU/0.01 ml), whether the organism was isolated in pure or mixed culture, and whether SCC was included as a complementary diagnostic criterion. Notably, the lowest diagnostic Se values (ranging from 0.44 to 0.69) were observed when only pure cultures were considered. This suggests that the higher diagnostic Se values were influenced by the inclusion of mixed cultures. In such cases, results showing only one or two colonies in mixed cultures should be interpreted with prudence. The detection of *S. aureus* might represent contamination rather than true IMI, considering its frequent presence on the skin of animals and humans, or from other environmental sources (36). In such cases, resampling is suggested to confirm results. These aspects highlight the importance of properly collected milk samples to avoid contamination that may lead to misclassification, limiting the veracity of culture-based diagnostic results. In contrast, diagnostic Se did not exceed 0.30 in the study evaluating Accumast, Mastatest, and Check-up, whereas diagnostic Sp remained consistently high, exceeding 0.99 (15). Another factor that can negatively impact diagnostic Se is the intermittent shedding of the pathogen, meaning that a single milk sample may not contain enough bacteria to be detected, particularly in chronic infections (37). In these situations, it is recommended to repeat sampling and freeze the obtained sample for 24 h to increase the likelihood of detecting the causative agent (5, 38).

In Colombia, *S. agalactiae* remains a prevalent pathogen, making its timely detection essential for controlling its rapid spread and reducing its impact on herd health (8). In field conditions, there is increasing concern over the lack of response to commonly used antibiotics for its treatment. The World Health Organization classified *S. agalactiae* as a medium-priority pathogen in its 2024 Bacterial Priority Pathogens List. This classification is due to its growing resistance to penicillin, one of the main antibiotics used to treat intramammary infections (39). Although inclusion in this list is guided primarily by human health impacts, the entry of *S. agalactiae* is linked mainly to neonatal sepsis-associated AMR. Nevertheless, resistance to penicillin is a relevant factor for antimicrobial usage in both human and animal health (40). The +tiDx system showed a diagnostic Se of 0.82 and a diagnostic Sp of 0.93, both relative to CRS, for the detection of this microorganism. Granja reported a diagnostic Se of 0.68 and a diagnostic Sp of 0.98 (35). Other diagnostic technologies (Accumast, Mastatest, and Check-up) did not report performance data for *S. agalactiae*, likely due to its low or absent prevalence in the study population.

The NAS group includes a wide variety of species that inhabit the skin of animals and humans, as well as the environment (41). Therefore, their detection in milk samples should be interpreted with caution due to the high likelihood of contamination. In the study by Dohoo et al., diagnostic Se values for NAS were generally low, ranging from 0.06 to 0.58, except under the criterion of detecting a single colony in a mixed culture, which yielded a diagnostic Se of 0.81 (16). For all definitions that incorporated SCC, regardless of the number of CFU/0.01 ml, diagnostic Se did not exceed 0.14. In our study, the diagnostic Se relative to CRS was 0.36, while the Brazilian-developed technology reported a higher diagnostic Se of 0.73 (35).The variability in diagnostic Se, diagnostic Sp remained consistently high across studies, with values above 0.95 (16, 35). Conventional microbiological culture techniques, such as SMC, may demonstrate low diagnostic sensitivity values for NAS and *S. aureus* when bacterial loads are low or shedding is intermittent. This limitation arises because intermittent or low-CFU/0.01 ml shedding can result in insufficient growth in culture, leading to false negative results, unless multiple samples are evaluated or repeated testing is performed (42). Furthermore, variability in sampling techniques and result interpretation further contributes to inconsistent culture outcomes, highlighting the user-dependent nature or traditional microbiological diagnosis (43). These findings reflect the diagnostic challenges associated with NAS detection. Balancing the identification of true infections and the avoidance of false positive results with different diagnostic systems due to contamination remains particularly complex.

The observed moderate concordance (C4: 0.54) between field-based and laboratory-based +tiDx evaluations was primarily linked to challenges in result interpretation rather than procedural issues, as observed by the accompanying veterinarians guiding the on-farm analysis process. While laboratory analyses were conducted by trained microbiology professionals, field evaluations were typically performed by farm personnel with little or no experience in this type of diagnostic testing. This gap became particularly evident in the interpretation of results, especially in samples involving more than one pathogen (mixed culture), where distinguishing color changes in the CromoSyS system proved somewhat difficult for farm users. These findings highlight the need to improve the result-reading algorithm to enhance its veracity in complex cases and to reinforce initial user training.

### 4.2 REASSURED comparison

The +tiDx diagnostic system demonstrated superior performance in REASSURED analysis compared to the SMC protocol across several key criteria, particularly in real-time connectivity, diagnostic Se, portability, and ease of use. Unlike SMC, which requires thoroughly trained personnel, specialized equipment, and laboratory infrastructure, +tiDx allows for result reading and interpretation without the need for specialized staff, as these tasks are supported by the +tiApp application, making it especially suitable for on-farm use. The system is user-friendly and can be operated by individuals with basic training, regardless of educational background (e.g., milkers, farm managers, or veterinarians).

The innovative syringe-like design minimizes exposure of the culture media to environmental conditions and simplifies sample inoculation, thereby reducing the risk of contamination (44–47), an essential advantage for POC diagnostics which was evidenced in this work with the decrease in contaminated samples observed through +tiDx analysis vs. SMC (21 vs. 33). Additionally, the incubation device also functions as a workstation, transforming the system into a portable laboratory. The unit can operate for up to 10 h without connection to a power source. This feature greatly facilitates the work of veterinarians providing technical support across multiple farms. It is also favorable for livestock companies with dairy operations in different regions and for associations that serve groups of dairy producers. The system can, as such, be used in various locations without the need for additional infrastructure (48).

The cost and turnaround time for results are significant limitations to the routine use of microbiological culture as a diagnostic tool, not only in Colombia but also in other countries (49, 50). From a cost perspective, +tiDx is a more cost-effective option, with an estimated price of USD 4.56 per sample, compared to the SMC, which in Colombia ranges from USD 5.70 (under company-subsidized schemes, which are not uniformly available to all producers) to USD 15.96. The cost of microbiological analysis in the country is a major limiting factor for its widespread use, along with the geographic distribution of specialized laboratories, which are often inaccessible for many producers. This limited accessibility contributes to delays in obtaining results. In response to these challenges, accessible POC tests like +tiDx offer a promising strategy to improve access and support timely decision-making in the field.

In conclusion, the +tiDx diagnostic system proved to be a valuable tool for the detection of IMI in subclinical mastitis cases, which are more prevalent, harder to detect, and responsible for sustained production losses in dairy herds. The system demonstrated adequate overall diagnostic performance for key Gram-positive pathogens. Its ability to provide faster, credible, on-farm results supported by +tiApp addresses key limitations associated with SMC, such as the need for specialized infrastructure and professionally trained personnel. Additionally, the affordability, portability, and ease of use of the system make it particularly well-suited for on-farm implementation. Although the interpretation of mixed infections remains a challenge, especially for users with minimal training, these findings underscore the importance of reinforcing initial training and refining the result-reading algorithm. Moreover, the variations found in concordances between laboratory and field evaluations too remark the importance of initial training to ensure correct result reading, and a necessary identification of the possible variables involved in these differences, along with further optimization of the evaluation process to reduce its impact in field performance. Still, +tiDx can serve as a support tool for dairy herd improvement programs by enabling early detection of infections, optimizing antimicrobial use, and facilitating more informed decision-making for animal health management.

### 4.3 Study limitations and future directions

This study has several limitations that should be considered. Gram-positive cocci that were catalase-positive but did not yield a positive reaction on the chromogenic agar for *Staphylococcus aureus* were classified as NAS, and catalase-negative Gram-positive cocci without a positive reaction on the chromogenic agar for group B *Streptococcus* were classified as other *Streptococcus* species. These categorizations were based solely on phenotypic characteristics and chromogenic media reaction, without confirmatory identification at the species or even genus level. We expect to verify our pathogen isolates in the future through DNA amplification in order to refine and confirm the CromoSyS identification system and its ability to correctly classify pathogens.

Furthermore, since the study focused exclusively on subclinical mastitis cases, it was not possible to evaluate the diagnostic performance of the system for detecting infections caused by Gram-negative bacteria. This will be addressed in a subsequent study involving clinical mastitis cases and a broader range of pathogens.

Finally, because SMC is an imperfect reference method, its use within the CRS likely introduced inherent uncertainty in our estimates, as true infection status could not be definitively established. Consequently, the diagnostic performance reported here may deviate from its true values. This limitation will be explored in a future analysis where statistical estimations considering a unique standard test, a CRS (as in this study), and Bayesian calculations will be compared and discussed.

## Data Availability

All relevant data is contained within the article: The original contributions presented in the study are included in the article/Supplementary material, further inquiries can be directed to the corresponding author/s.
