# Supplementary material for: Validation of +tiDx: a point-of-care diagnostic system for Gram-positive bovine mastitis
Source: Front Vet Sci. 2026 Feb 11;13:1766686. doi: 10.3389/fvets.2026.1766686 (PMC12932151; doi:10.3389/fvets.2026.1766686)
Supplement: Supplementary file 1 [file Image_1.pdf]

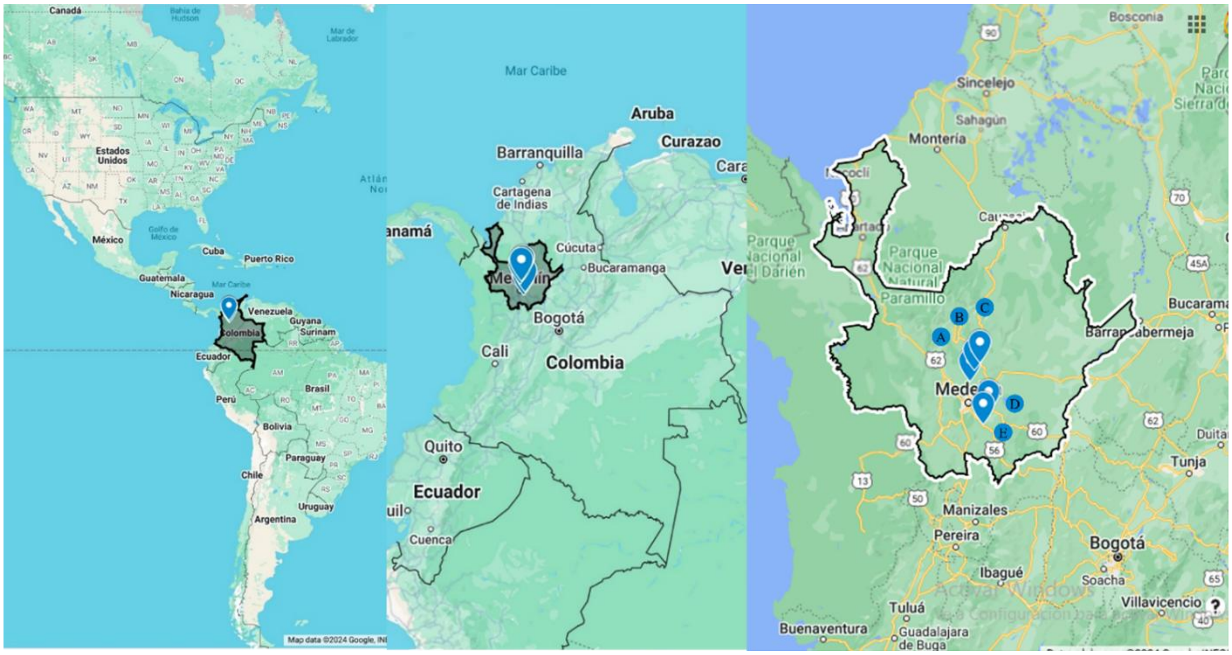

Supplementary Figure 1. Geographical context for the location of dairy farms sampled**.** 1. Location of Colombia in the context of North and South America; 2. Location of Antioquia in the context of Colombia; 3. Location of each municipality sampled in the context of Antioquia: A) San Pedro de los Milagros; B) Entrerríos; C) Santa Rosa de Osos; D) Rionegro; E) La Ceja.
